# Understanding Number Line Estimation in Williams Syndrome and Down Syndrome

**DOI:** 10.1007/s10803-019-04268-7

**Published:** 2019-11-08

**Authors:** V. Simms, A. Karmiloff-Smith, E. Ranzato, J. Van Herwegen

**Affiliations:** 1 School of Psychology, Ulster University, Coleraine, UK; 2 Department of Psychology, Kingston University London, London, UK; 3 Department of Psychology and Human Development, UCL Institute of Education, London, UK

**Keywords:** Williams Syndrome, Down Syndrome, Number line estimation, Visuo-spatial skills, Number familiarity

## Abstract

Previous studies suggest that tasks dependent on the mental number line may be difficult for Williams Syndrome (WS) and Down Syndrome (DS) groups. However, few have directly assessed number line estimation in these groups. The current study assessed 28 WS, 25 DS and 25 typically developing (TD) participants in non-verbal intelligence, number familiarity, visuo-spatial skills and number line estimation. Group comparisons indicated no differences in number line estimation. However, the WS group displayed difficulties with visuo-spatial skills and the DS group displayed difficulties with number familiarity. Differential relationships between number line estimation and visuo-spatial/number familiarity skills were observed across groups. Data is discussed in the context of assessment of skills in neurodevelopmental disorders.

Mathematical skills are essential for everyday functioning, for example, being able to calculate change in a shop, read timetables and make purchasing decisions. Previous literature consistently reports that individuals with Williams Syndrome (WS), a relatively rare chromosomal disorder (1 in 20,000 live births) caused by deletion of the long-arm of chromosome 7q11.23, and Down Syndrome (DS), a more frequently occurring neurodevelopmental disorder (1 in 800 live births), caused by a trisomy or mosaicism of chromosome 21, have specific difficulties with mathematical processing (e.g. Ansari et al. 2003; Libertus et al. 2014; Paterson et al. 2006; O’Hearn and Landau 2007). These difficulties have significant ramifications for general learning, school achievement and also for independent living (Stancliffe and Lakin 2007).

Although there is a great deal of diversity within neurodevelopmental disorders (Van Herwegen et al. 2011; Karmiloff-Smith et al. 2016), both individuals with WS or DS have been reported to have overall similar intelligence quotient (IQ) scores, generally between 50 and 70 (Martens et al. 2008; Mervis et al. 2003). Yet, these two groups show different uneven cognitive profiles, in that individuals with WS have better language and short-term memory abilities compared to their visuo-spatial difficulties (Martens et al. 2008; Mervis et al. 2003; Van Herwegen et al. 2011). In contrast, individuals with DS show poorer language abilities and short-term memory abilities compared to their visuo-spatial skills (Grieco et al. 2015; Silverman 2007). The varying overlapping strengths and difficulties in these disorders’ phenotypes allow for useful cross-syndrome comparisons and the examination of the impact of different underlying difficulties on phenotypical outcomes (Ball and Karmiloff-Smith 2015; Van Herwegen and Karmiloff-Smith 2015).

As mathematics is a multi-componential subject, there are numerous cognitive skills that contribute to successful learning in this domain, such as language skills and visuo-spatial processing, areas of relative weakness in the two aforementioned neurodevelopmental disorders. In addition, a major focus of recent research has been the influence of numerical representations, or how numerical information is represented in individuals’ minds, on more complex mathematical achievement (e.g. Inglis et al. 2011; Purpura and Simms 2017). The dominant theory suggests that numbers are stored along a mental number line, with small numbers associated with the left-hand-side of space and large numbers associated with the right (Dehane 1997). Within numerical cognition research a number of different tasks have been used to assess these representations, such as symbolic and non-symbolic comparison tasks, approximate addition tasks and number line estimation. However, there is extensive debate around the precise nature of the construct that these tasks may measure (e.g. Cohen and Quinlan 2017; Siegler 2016).

## Number Line Estimation

A task that has gained substantial attention is the number line estimation task (c.f. Siegler and Opfer 2003). In this task, participants are asked to position a series of numbers on blank number lines (number-to-position task) or to state what number should be on a specific position on the number line (position-to-number task). Number lines may indicate the scale of the line (bounded, e.g. 0–10, 0–100) or may be unbounded (with no scale demarcations). It has been consistently observed that with development, participants become more accurate at positioning or identifying numbers on the number line, quantified as decreasing error rates in their estimations (Siegler and Booth 2004; Praet and Desoete 2014). This developmental change is also reflected in a shift in pattern of estimations across the number line, from being best explained by a logarithmic function (with small numbers compressed at the left hand side of the number and larger numbers widely spread towards the right) to a linear function (with numbers being evenly spread across the number line). This developmental change is scale dependent, with changes occurring on small scales (e.g. 0–10, 0–20) before larger scales (e.g. 0–100, Muldoon et al. 2013). This pattern of development is consistent with Siegler’s Overlapping Waves Model (1996).

A widely replicated finding is that performance on the number line estimation task (as quantified by either the linear fit of the estimation profile or accuracy on the task) is related to more complex mathematical processing (such as accuracy in addition performance or scores on standardised mathematics tests; e.g. Friso-van de Bos et al. 2015; LeFevre et al. 2013; Muldoon et al. 2013). In addition, interventions that have focused on increasing accuracy or linearity of performance on the number line estimation task have observed both increased accuracy/linearity on the task and also positive transfer to increased accuracy in performance in general mathematics tasks (e.g. Dackermann et al. 2013; Siegler and Ramani 2009). Thus, number line estimation is related to more general mathematical achievement, and the data from intervention studies suggest that this relationship is causal.

However, current debate focuses on whether number line estimation provides direct insight into individual’s internal numerical representations (as suggested by Siegler 2016; Siegler and Braithwaite 2017), with different interpretations suggesting that number line estimation does not provide “a window onto mental representations of quantity” (Cohen and Quinlan 2017). Instead, it is suggested that performance on this task more closely reflects constraints of the task itself. Therefore, any findings from studies should be interpreted carefully and additional explanations should be discussed, including the influence of a variety of cognitive skills on performance.

## Factors Related to Number Line Estimation Performance

A growing body of evidence suggests that numerous additional skills are important for success on the number line estimation task, specifically number system knowledge (e.g. Muldoon et al. 2013) and visuo-spatial skills (e.g. Simms et al. 2016). Number familiarity is an important domain of knowledge for number line estimation in typically developing children. Number familiarity can be assessed by a variety of tasks, such as reciting number word lists, digit recognition or enumeration of sets of objects. LeFevre et al. (2013) observed that number system knowledge (as assessed by a standardised numeration test, that included trials such as ordering) was an independent predictor of number line estimation skills in 9–10 year-old. In addition, Muldoon et al. (2013) reported that children’s ability to recite the number word list and enumerate sets of 10 and 20 objects was related to number line estimation performance. More specifically, children who could recite the number word list to a higher value, or could more accurately count sets of objects, displayed estimation patterns that were best described by linear functions.

There is also a wide body of evidence that indicates a strong relationship between visuo-spatial skills and mathematical achievement in general (Assel et al. 2003; Mix and Cheng 2012, Gilligan et al. 2017; Wai et al. 2009). Just as mathematics is multi-componential, so too are visuo-spatial skills (Uttal et al. 2013). Specifically, a number of previous studies have investigated the relationship between visuo-spatial processing and number line estimation. Thompson et al. (2013) established that there was a significant relationship between mental rotation skills (i.e., being able to generate a mental representation of an object and rotate this in mental space) and number line estimation in an adult sample, with higher rotation skills associated with better number line estimation performance.

Crollen and Noël (2015) examined the relationship between a composite visuo-spatial score, comprising picture copying performance and a parent report of visuo-spatial skills, and number line estimation in 9½ year-olds. Children with heightened visuo-spatial skills performance had better number line estimation performance. LeFevre et al. (2013) assessed visuo-spatial skills in 5-to-9-year-olds by administering a subscale of a standardised test (Analogy: Cognitive Intelligence Test-Nonverbal, Gardner 2000) that assesses a range of visuo-spatial skills, including mental rotation. Scores on this task were combined with performance on a child-friendly computerised visuo-spatial working memory test. A moderate, significant relationship was observed between visuo-spatial skills and number line estimation.

Additional evidence from Gunderson et al. (2012) emphasised the concurrent and predictive significant relationship between mental rotation skills and number line estimation in 7-year-old, even after controlling for additional factors. Importantly, in a longitudinal study included within the same paper with 5–8-year-old, a combination of mental rotation and mental transformation skills (as measured by 3D block design) fully mediated the relationship between number line estimation and calculation skills. Simms et al. (2016) also indicated that in 8–10 year-olds the relationship between mathematical achievement and number line estimation was fully mediated by visuo-motor skills (measured by a design copying task) and visuo-spatial processing (as measured by a mental rotation and disembedding task). Therefore, there is building evidence that visuo-spatial skills are significantly related to number line estimation, and in some scenarios these skills *at least* partially mediate the relationship between number line estimation and mathematical achievement.

## Number Line Estimation in WS and DS

There are very few studies that specifically investigate number line estimation in individuals with WS or DS. In general terms, it has been reported that individuals with WS may have impairments of the mental number line (O’Hearn and Luna 2009). In addition, O’Hearn and Landau (2007) reported that individuals with WS performed similarly in a standardised mathematical test when compared to mental age (MA) matched controls. However, when investigating specific items on this test, individuals with WS displayed difficulties in number comparison questions that are thought to rely on the mental number line. In contrast, and in line with known verbal strengths, individuals with WS performed better than controls on number word reading questions.

Only two studies have carried our cross-syndrome comparisons on tasks thought to be dependent on *mental number line representations*. Paterson et al. (2006) carried out a series of experiments to assess numerical representations in WS and DS. They observed that WS infants performed to the same level as MA matched controls on a preferential looking paradigm that manipulated the numerosity of presented visual stimuli. In contrast, control infants outperformed DS participants. The same paper also reported experimental results with older DS, WS, and control participants. In quantity comparison tasks that were thought to rely on the mental number line, DS and control participants outperformed those with WS. Therefore, although there does appear to be a specific difficulty in the utilisation of the mental number line in both WS and DS, there are syndrome specific differences. In addition, Karmiloff-Smith et al. (2012) contrasted performance between DS and WS infants and toddlers on small exact, and large approximate, quantity discrimination performance. This amalgamation of studies indicated a double dissociation in these skills across syndromes, with the WS group succeeding in small exact, but not large approximate, discrimination and vice versa for the DS group. However, it is important to note that both Paterson et al. and Karmiloff-Smith et al. (a) included assessments of infants and toddlers and (b) did not directly measure number line estimation performance.

Only one previous paper has directly assessed number line estimation skills in WS groups. In this intervention study, Opfer and Martens (2012), assessed performance on a 0–1000 number line, it was observed that improvements in estimation accuracy were associated with increasing age. A brief feedback intervention also led to increased accuracy (i.e. the average difference between the actual positions of numbers and their estimated points), but no concurrent improvement in function of the estimations (i.e. the spread of the estimations across the number line) was observed. The function of estimations (i.e. logarithmic or linear distributions) are commonly interpreted as a marker of the internal representation of the number system. Therefore, the authors argued that *representational* change does not occur in WS groups, either through development or in response to intervention. It should be highlighted that the number line estimation task used in this study was well beyond the bounds of number system knowledge for a WS group and this may have impacted on the observed results. No mathematical achievement measure was administered. Therefore, it remains an open question as to whether number line estimation is related to mathematical achievement in a WS sample.

Similarly, only one study to date has used a number line estimation task with a DS sample (Lanfranchi et al. 2015). In this study the DS group performed to the same level of accuracy as a typically developing MA matched group on a 0–10 scale, but displayed poorer performance than a chronological age (CA) matched group (Lanfranchi et al. 2015). In contrast, the DS group outperformed a MA matched group on the 0–100 scale, but their performance was also worse than a CA matched group. Similar patterns were observed for ${\text{R}}^{ 2}_{\text{Lin}}$ values. Although numerical abilities were assessed within this study, correlations were not conducted between number line estimation and the outcome measure. Therefore, similar to WS, it is not known if number line estimation is related to mathematical achievement in DS samples and whether number line performance relates to their visuo-spatial abilities or number familiarity.

Comparison of relationships between number line task performance, mathematical abilities, visuo-spatial abilities and number familiarity in syndromes that have similar mathematical abilities, but specific difficulties in visuo-spatial (WS) and number familiarity (DS), will allow a better understanding of what cognitive abilities are measured by the number line task. In addition, understanding these relationships in WS and DS will allow for the development of better mathematical intervention programmes for these neurodevelopmental disorders

## The Current Study

The current study assessed number line estimation performance in WS, DS and a typically developing (TD) MA matched group, using developmentally appropriate measures. Recent meta-analysis has concluded that the number line estimation task should be utilised as an assessment tool of numerical understanding (Schneider et al. 2018). Therefore, it is essential to assess if this task assesses the same types of skills in different population samples. This study addressed the following research questions:

1. Are there significant differences between WS and DS in number line estimation performance (as measured by error rates and ${\text{R}}^{ 2}_{\text{Lin}}$ values)?
2. Is number line estimation related to mathematical achievement in WS, DS and TD groups?
3. Are visuo-spatial skills and number familiarity associated with number line estimation performance in the three groups?
4. What factors (visuo-spatial and/or number familiarity) predict number line estimation performance in WS, DS and TD groups?

## Method

### Participants

Twenty-eight participants with WS (18 females) aged 8;00 to 52;25 years old were recruited via the WS Foundation. All participants with WS had a positive diagnosis for WS using the genetic fluorescent in situ hybridisation (FISH) test confirming the genetic deletion implicated in WS, in addition to a clinical diagnosis for WS. None of the WS participants had a comorbid diagnosis. Twenty-five participants with DS aged 8;06 to 49;17 (12 females) were recruited via DS support groups across the South-East of the UK. All of the DS participants had a genetic mutation on chromosome 21. Twenty-five typically developing (TD) children (10 females) whose IQ fell within the two neurodevelopmental groups scores were included in the study. TD children were aged between 4:5 and 10:2 years old. All participants had English as a first language and none of the TD participants had a diagnosis for a learning difficulty.

### Materials and Procedure

Ethical governance was provided by the Kingston University’s Ethics Committee. All parents/guardians were provided with in depth information sheets and provided informed written consent. Verbal assent was provided by all participants.

*Mathematical Achievement* was measured using the WIAT-II Mathematics subscale (Wechsler 2005). Participants were required to complete a series of tasks with increasing complexity beginning with simple counting, progressing to basic arithmetic and more complex problem solving. The test was administered following standardised procedures; once the participant produced six consecutive incorrect answers the test administration was terminated. Raw scores were used in analysis (potential range of scores: min = 0, max = 54).

*General Intelligence* was assessed using Raven’s Coloured Progressive Matrices (Raven 2008). In this task, participants were presented with 36 trials. Each trial displayed a pattern with a missing “piece”; participants were required to indicate from a set of images which “piece” completes the pattern (either by pointing or saying the number of the piece). Raw scores were used in analysis (potential range of scores: min = 0, max = 36).

*Visuo*-*spatial Abilities* were measured using the Pattern Construction Task from the British Ability Scales-II (Elliott et al. 2008). Participants were required to replicate images presented to them by the experimenter, first using tiles and then progressing to more complex patterns responding using 3D blocks, following standardised instructions. Ability scores were used in the analyses, due the application of starting rules (potential range of scores: min = 0, max = 220).

#### Number Line Estimation

The number line estimation task was administered following the instructions of Siegler and Opfer (2003). Children were presented with the number line estimation task on two scales, 0–10 and 0–100. The 0–10 scale was completed first. Before beginning the test trials a practice trial was administered. Participants were presented with a blank number line, the experimenter stated “Zero goes here and 10 goes here, where would 5 go?” The experimenter then marked the position of 5 on the number line. The experimenter stated “Now it’s your turn, if zero goes here and 10 goes here where would x go?” This was repeated for all values that were to be estimated. The same procedure was followed for the 0–100 scale, the practice item was “50”. Participants were asked to position eight numbers (1,2,3,4,6,7,8,9) on the 0–10 scale and 13 numbers on the 0–100 (3,6,12,17,21,29,33,48,57,61,77,83,96) scale. Further details on scoring this task is included in the preliminary data computation section.

#### Number Familiarity

These skills were assessed using a variety of counting and recognition tasks. Counting tasks were designed following Paterson et al. (2006). Participants were asked to count up to 20, beginning at 1. The highest number that the participant reached was recorded (max = 20). Participants were also asked to count backwards from 20, the number of correct count steps that the participant made was recorded (max = 20). To assess more advanced counting skills participants were requested to count on from 25 to 35 and were awarded one point if they successfully completed the task. In order to assess digit recognition, children were presented with cards with the digits from 1 to 20. These cards were presented in a random order. Participants were asked to verbally name the presented digits. The total number of correctly identified cards was recorded (max = 20). A number familiarity score was calculated by averaging the percentage accuracy across the four tasks (potential range of scores: min = 0%, max = 100%).

### Procedure

Parents were provided with detailed information about the project and provided written consent, whilst verbal assent was obtained from all participants. This project had received favourable opinion from the Kingston University’s Ethics Committee.

The tasks were presented in random order to participants and they were assessed one-on-one with a researcher in a quiet room at the university. The total testing session took about 1 h and breaks were taken as often and for long as required by the participant.

#### Preliminary Data Computation

In order to analyse the number line estimation data some preliminary calculations were conducted. To generate percent absolute error (PAE) scores for each participant the following calculation was conducted for each estimation on both the 0–10 and 0–100 scales.

$$ {\text{PAE}}\, = \,\frac{{\left( {{\text{Value of estimated point}} - {\text{ number to be estimated}}\, \times \, 100} \right)}}{{{\text{Number}}\,{\text{line scale}}}} $$

Then, an average PAE score for each participant for each scale was calculated. In addition, curve estimation was carried out in SPSS for each participant on each scale separately. The independent variable was the value of the numbers to be estimated and the dependent variable was the value of the estimated points. Curve analysis generated ${\text{R}}^{ 2}_{\text{Lin}}$ values for further analyses.

## Results

Proceeding the preliminary data computation, it was noted that one participant on the 0–100 scale only responded to three out of 13 estimation points. Therefore, they were excluded from analyses on the 0–100 scale.

Table 1 summarises descriptive statistics for the three groups (TD, WS and DS).

**Table 1:** Descriptive statistics

	TD		WS		DS	
	N	M (SD)	N	M (SD)	N	M (SD)
Age	25	74.0 (18.8)	28	244.7 (160.9)	25	258.4 (128.6)
Non-verbal IQ	25	17.3 (5.8)	28	15.6 (4.5)	25	15.8 (6.4)
Mathematical achievement	25	11.0 (7.2)	28	8.6 (3.7)	25	8.2 (3.0)
Visuo-spatial skills	21	103.1 (26.2)	28	65.7 (31.0)	22	84.2 (22.5)
Number familiarity	25	76.2 (23.5)	28	86.1 (17.3)	25	67.6 (21.9)
PAE 0–10	25	13.8 (9.4)	28	16.3 (8.8)	25	18.7 (12.6)
PAE 0–100	24	22.8 (13.4)	26	22.1 (9.0)	23	25.6 (12.1)
${\text{R}}^{ 2}_{\text{Lin}}$ 0–10	25	.8 (.3)	28	.7 (.3)	25	.7 (.3)
${\text{R}}^{ 2}_{\text{Lin}}$ 0–100	24	.5 (.4)	26	.5 (.3)	23	.3 (.3)

Univariate analysis of variance (ANOVA) revealed a significant main effect of group for age, *F*(2,75) = 18.2, *p *< .001, *n*_p_^2^ = .3, with the TD group being significantly younger than both the WS (MD = − 170.6 months, *p* < .001) and DS groups (MD = − 184.4 months, *p* < .001). There was a significant main effect of group on visuo-spatial skills, *F*(2,68) = 11.4, *p* < .001, n_p_^2^ = .3, with the TD group having significantly better visuo-spatial skills than the DS group (MD = 18.9, *p* = .026) and the DS having significantly better visuo-spatial skills than the WS group (MD = 18.6, *p* < .001). There was also a significant main effect of group on number familiarity, *F*(2,73) = 5.2, *p* = .008, n_p_^2^ = .1, with the WS group scoring significantly higher in the number familiarity task than the DS group (MD = 18.6, *p *= .002). There was no significant main effect of group for non-verbal IQ or mathematical achievement. These findings confirm different cognitive profiles for individuals with WS and DS despite being matched on overall IQ.

### Differences in Number Line Estimation

Four univariate ANOVAs were conducted for each number line estimation metric on each scale. The independent variable was the number line estimation metric (PAE or ${\text{R}}^{ 2}_{\text{Lin}}$) and the fixed factor was group (WS, DS, and TD). There was no significant main effect of group for either of the number line estimation task metrics or for either of the scales (0–10 PAE: F(2,75) = 1.414, p = .250; 0–100 PAE: F(2,70) = .627, p = .537; 0–10 ${\text{R}}^{ 2}_{\text{Lin}}$: F(2,75) = .725, p = .488; 0–100 ${\text{R}}^{ 2}_{\text{Lin}}$: F(2,70) = 1.882, p = .160). In addition, results of Bayesian ANOVAs indicated anecdotal to moderate evidence for the null hypothesis, that there were no group differences in number line estimation task performance (0–10 PAE: BF_10_ = .194; 0–100 PAE: BF_10_ = .334; 0–10 ${\text{R}}^{ 2}_{\text{Lin}}$: BF_10_ = .328; 0–100 ${\text{R}}^{ 2}_{\text{Lin}}$: BF_10_ = .188). Therefore, there was no significant difference between groups in their performance on the number line task, independent of metric or scale, and these frequentist statistics were supported by Bayesian analyses.

### Relationship Between Number Line Estimation and Mathematical Achievement

Table 2 summarises the results of Pearson’s correlations between number line estimation metrics and mathematical achievement for each group on each scale

**Table 2:** Pearson’s correlations between number line metrics and mathematical achievement

	TD	WS	DS
PAE 0–10	− .644**	− .404*	− .531**
PAE 0–100	− .772**	− .577**	− .658**
${\text{R}}^{ 2}_{\text{Lin}}$ 0–10	.497*	.492**	.488*
${\text{R}}^{ 2}_{\text{Lin}}$ 0–100	.785**	.548**	.423*

*p < .05, **p < .01

Pearson’s correlations indicated that, for all groups and for both scales, number line estimation was significantly correlated with mathematical achievement. Decreasing error rates and increasing ${\text{R}}^{ 2}_{\text{Lin}}$ values were significantly associated with increased mathematical achievement.

### Relationship Between Visuo-spatial Skills and Number Familiarity with Number Line Estimation Performance

As similar patterns were observed between both PAE and ${\text{R}}^{ 2}_{\text{Lin}}$ values further analyses focused on PAE values only. Table 3 summarises the results of Pearson’s correlations between number line estimation and visuo-spatial skills for each group on each scale. Table 4 summarises the results of Pearson’s correlations between number line estimation and number familiarity for each group on each scale.

**Table 3:** Pearson’s correlations between number line estimation and visuo-spatial skills

	TD	WS	DS
	r	r	r
PAE 0–10	− .560*	− .189	− .556*
PAE 0–100	− .708**	− .299	− .478*

*p < .05, **p < .01

**Table 4:** Pearson’s correlations between number line estimation and number familiarity

	TD	WS	DS
	r	r	r
PAE 0–10	− .508*	− .593*	− .367
PAE 0–100	− .807**	− .509*	− .436*

*p < .05, **p < .01

Table 3 indicates that for both the TD and DS group visuo-spatial skills and number line estimation were significantly correlated for both the 0–10 and 0–100 scales. Heightened visuo-spatial skills were associated with decreased error rates in number line estimation. No significant correlations were observed between visuo-spatial skills and number line estimation in the WS group.

Table 4 indicates that for both the TD and WS group number familiarity and number line estimation was significantly correlated for both the 0–10 and 0–100 scales. Heightened number familiarity was associated with decreased error rates in number line estimation. No significant correlation was observed between number familiarity and number line estimation on the 0–10 scale in the DS group. However, a significant correlation was observed between number familiarity and number line estimation on the 0–100 scale in the DS group.

### Predictors for Number Line Estimation Performance in WS, DS and TD Group

Separate linear regressions for both number line scales (0–10 and 0–100) and each group (TD, WS, DS) were conducted. These results are summarised in Tables 5 and 6. The dependent variable was PAE and the predictor variables were visuo-spatial skills and number familiarity.

**Table 5:** Linear regressions for 0–10 scale per group

	TD	WS	DS
	r	r	r
Adjusted R^2^	.379*	.299*	.237*
Predictors			
Visuo-spatial skills	− .273	.006	− .534*
Number familiarity	− .461	− .594*	− .038

*p < .05, **p < .001

**Table 6:** Linear regressions for 0–100 scale per group

	TD	WS	DS
	r	r	r
Adjusted R^2^	.725**	.218*	.222*
Predictors			
Visuo-spatial skills	− .302	− .144	− .366
Number familiarity	− .647*	− .461*	− .295

*p < .05, **p < .001

Table 5 indicates that significant models were produced for all groups on the 0–10 scale data. Number familiarity was a significant unique predictor for the WS group, in contrast visuo-spatial skills were a significant unique predictor for the DS group. There were no significant unique predictors for the TD group.

Table 6 indicates that significant models were produced for all groups on the 0–100 scale data. Number familiarity was a significant unique predictor for both the WS and TD groups. There were no significant unique predictors for the DS group.

## Discussion

Overall, this study established that, after matching for mental age across groups, performance on the number line estimation task was similar for TD, DS and WS participants on both the 0–10 and 0–100 scale. Participants with WS displayed significant difficulties with visuo-spatial processing, whilst those with DS displayed significant difficulties with number familiarity. Number line estimation performance (on both the 0–10 and 0–100 scale) was significantly associated with mathematical achievement for all groups. Number familiarity was a unique predictor of number line estimation performance (on both 0–10 and 0–100 scale) for WS participants, whilst visuo-spatial skills was a unique predictor of DS participants’ performance on the 0–10 scale.

There has been only two cross-syndrome comparison studies focusing on basic numerical skills in WS and DS (Karmiloff-Smith et al. 2012; Paterson et al. 2006). Importantly, these previous studies only utilised *non*-*symbolic stimuli* in order to understand participants’ mental number line. Developmentally, DS participants presented with quantity representation difficulties in infancy when compared to TD and WS participants, in contrast, in later childhood and adulthood WS participants displayed quantity representation difficulties (Paterson et al. 2006). Karmiloff-Smith et al. (2012) indicated more subtle cross-syndrome differences, with WS group struggling with small, exact quantity comparison and DS group displaying difficulties with large, approximate quantity comparison. The current study indicates that, when using *symbolic stimuli* there are no differences in performance between WS, DS and mental-age-matched TD groups.

In addition, and in contrast to Opfer and Martens (2012) who identified group differences between WS and TD groups when utilising a scale that was well beyond the numerical knowledge of most WS groups (0–1000), the current study reported no group differences. This may be due to the use of 0–10 and 0–100 scales in this study. These results highlight the importance of developmentally appropriate task selection and the implications for the interpretation of findings in terms of the identification of strengths and weaknesses associated with specific neurodevelopmental disorders.

This is the first study to *directly* compare number line estimation performance across WS, DS and TD participants. By utilising the same tasks across the two disorders, which have very different cognitive profiles, this study not only provides important information about performance differences, but also allows us to assess the impact of different low-level difficulties on outcome measures (Ball and Karmiloff-Smith 2015; Van Herwegen and Karmiloff-Smith 2015). Replicating previous research findings we established that WS participants displayed relative strengths in a verbally based number familiarity task (Ansari et al. 2003), but relative weaknesses in visuo-spatial processing (Farran and Jarrold 2003). The reverse was true for the DS group (Sella et al. 2013). What is striking is that preserved areas of cognition in both syndromes (WS = number familiarity; DS = visuo-spatial skills) predict performance on the number line estimation task. This suggests that individuals are harnessing the skills that they possess to complete the task, thus number line estimation in WS and DS draws upon different banks of skills. The lack of relationship between visuo-spatial skills and number line estimation in WS supports previous findings that indicated a lack of development of proficient visuo-spatial processing skills in this group (Van Herwegen et al. 2011).

These findings have both important theoretical and applied implications. Contributing to the debate surrounding what precisely the number line estimation task measures, these data indicate that task performance may reflect different task performance strategies in different populations. Therefore, it is quite impossible to suggest that this task is a pure measure of numerical representations (as suggested by Siegler 2016). Adding to the commentary provided by Cohen and Quinlan (2017) who emphasised the importance of task constraints on performance and interpretation, we would suggest that the task also reflects the general cognitive skills of the participant sample. As identified in the current study, when assessing atypical populations, we cannot simply make the inference that performance on such a measure relies on the same cognitive skills as typically developing groups.

This leads us to the application of our findings. There has been a call to suggest that number line estimation should be used as a tool to assess basic numerical understanding (Schneider et al. 2018). The results from the current study would suggest this is approached with caution in terms of widespread application across different populations. Our data indicate that the use of the number line estimation task measures very different skill sets in WS and DS, and therefore may not provide accurate assessment of these groups basic number skills.

There are many strengths of this study. By recruiting a relatively large sample size for neurodevelopmental research, the conclusions drawn from this paper increases confidence in the results. The use of developmentally appropriate tasks also increases the validity of the study. No significant group differences were found in number line estimation performance when using inferential statistics. However, the lack of statistically significant findings are not informative. Therefore, the use of Bayesian statistics in the current study enables us to conclude that there is anecdotal to moderate evidence for the null hypotheses (that group differences in number line estimation performance do not exist). A limitation of this study is that we did not record strategies that were used by participants whilst completing the number line estimation task, perhaps by using eye tracking technology. These data would have furthered our understanding of the potential differential mechanisms used to complete the task.

In conclusion, the findings from the current study indicate that it is important to use developmentally appropriate tasks when assessing performance in neurodevelopmental disorders as this may explain previously identified group differences in performance (i.e. in this scenario a number line scale that is within the realms of number knowledge of the participants). The current study also highlights that caution should be applied when assessing, and interpreting, performance from participants with neurodevelopmental disorders as similar performance can be driven by different cognitive skills. Our findings also indicate that performance on a number line task may draw on a variety of complex cognitive mechanisms.
